# Development and definition of a simplified scoring system in patients with multiple myeloma undergoing stem cells transplantation on standard computed tomography: myeloma spine and bone damage score (MSBDS)

**DOI:** 10.1186/s40644-020-00306-1

**Published:** 2020-04-28

**Authors:** Alberto Stefano Tagliafico, Liliana Belgioia, Alessandro Bonsignore, Alessio Signori, Matteo Formica, Federica Rossi, Michele Piana, Daniela Schenone, Alida Dominietto

**Affiliations:** 1 Department of Health Sciences, University of Genoa, Via A. Pastore 1, 16132 Genoa, Italy; 2 Ospedale Policlinico San Martino, Largo R. Benzi 10, 16132 Genoa, Italy; 3 Dipartimento Di Scienze Chirurgiche e Diagnostiche Integrate – Disc, University of Genoa, Via A. Pastore 1, 16132 Genoa, Italy; 4 Dipartimento Di Matematica – Dima, University of Genoa, via Dodecaneso 35, 16146 GENOVA Genoa, Italy; 5 CNR – SPIN, Genoa, Italy

**Keywords:** Multiple myeloma, Computed tomography, Score, Fracture, Risk, Stem cell transplantation

## Abstract

**Background:**

In clinical practice, there is the need to optimize imaging usage in MM patients. Accordingly, the aim of this paper was to develop a simple computed tomography (CT) scoring method for MM, able to shorten and simplify the interpretation time with good intra- and inter-reader reliability. This method, named MSBDS (Myeloma Spine and Bone Damage Score) was developed with the final aim to use standard total-body CT in the routine practice of MM centres as a complement of standard evaluations in patients undergoing stem cells transplantation.

**Methods:**

We used a widely accepted consensus formation method and literature research during three structured face-to-face meetings specifically designed to combine opinions from a group of experts with proven experience in multiple myeloma care and/or musculoskeletal CT to facilitate the consensus on the field of study topics and the contents of the MSBDS score. Seven practical requisites for the MSBDS score were agreed. A total of 70 MM patients (mean age, 60 years ±9.2 [standard deviation]; range, 35–70 years) undergoing total-body CT was included to develop MSBDS scores. Patients data were already stored in the Radiological database for other Research studies IRB approved (054/2019). Readers to test the MSDMS were radiologists and clinicians involved in MM care or expert in bone damage scores with different level of experience in musculoskeletal and total body CT. Readers were blinded to the clinical data of the patients.

**Results:**

The MSBDS scores based on the consensus work described above and literature analysis was finalized. MSBDS is based on an additive scale with assessment of a total body CT with the bone window one time and includes indicators of structural bone damage and instability or fracture risk. The total score is given by the sum of item scores for abnormalities detected. Its values range from 0 (minimum) to values > 10 where 10 is represented by high-risk patients. In high-risk patients immediate surgical or radiation oncologist consultation is suggested.

**Conclusions:**

The MSBDS descriptive criteria are easy, highly reproducible and can be considered as a strong base for harmonizing total body CT interpretation in multiple myeloma patients undergoing stem cell transplantation.

## Introduction

Abnormal production of monoclonal immunoglobulin M component of plasma cells and bone marrow increase of plasma cells is the typical characteristic of multiple myeloma (MM). The bone lesions of myeloma are caused by the proliferation of tumour cells from a single clone and the activation of osteoclasts that destroy the bone [1]. Indeed, bone disease reduces patients’ quality of life increasing both morbidity and mortality, therefore imaging is crucial in the management of patients with MM. Imaging is important to detect bone lesions requiring immediate start of therapy or follow-up after treatment, to predict the risk of early progression from smouldering MM (SMM) to active disease, to identify sites of extra-medullary disease and to identify sites of bone disease at potential risk of pathologic fractures or neurologic complications [2]. According to recent staging systems for MM, in patients with new diagnosis, a correct treatment approach and evaluation of prognostic factors rely also on lesions identification on Magnetic Resonance Imaging (MRI), Computed Tomography (CT) or PET/CT [3]. Indeed, the role of conventional radiography, the standard of care for many years, is being replaced by more sensitive methods. According to large retrospective studies PET/CT [4] and whole-body low-dose CT (WBLDCT) [5], compared to conventional radiography are able to detect the presence of active disease in up to 25 to 40% of cases negative at conventional radiography [2]. At diagnosis the incorporation of new imaging modalities (WBLDCT and PET/CT) for accurate diagnostic purposes is recommended with a grade A recommendation [2]. However, there is still considerable heterogeneity in clinical practice regarding imaging usage in MM [2] and the updated International Myeloma Working Group (IMWG) criteria now allow for the use of computed tomography (CT), low-dose whole-body CT, and positron emission tomography with computerized tomography (PET-CT) to diagnose lytic bone disease in MM. However, there is still high variability in the choice between various imaging methods [4] and high variability in image interpretation with suboptimal agreement among readers in CT image interpretation to detect even clinically significant small lytic lesions. Therefore, in clinical practice, there is the need to optimize imaging usage in MM patients. Accordingly, the aim of this paper was to develop a simple CT scoring method for MM, able to shorten and simplify the interpretation time with good intra- and inter-reader reliability. This method, named MSBDS (Myeloma Spine and Bone Damage Score) was developed with the final aim to use standard total-body CT in the routine practice of MM centres as a complement of standard evaluations in patients undergoing stem cells transplantation.

## Methods

We used a widely accepted consensus formation method and literature research, i.e. the nominal group technique (2), during three structured face-to-face meetings specifically designed to combine opinions from a group of experts with proven experience in multiple myeloma care and/or musculoskeletal CT to facilitate the consensus on the field of study topics and the contents of the MSBDS score.

The following requisites for the MSBDS score were agreed: i) easiness in learning by non-expert musculoskeletal radiologists; ii) easiness of use by means of conventional total-body CT scans iii) lack of any requirement for high-end and/ or proprietary technology, i.e. lack of necessity to have low-dose whole body CT or whole body MRI; iv) informativeness in the assessment of bone status compared with standard radiography (skeletal survey), MRI and PET-CT; v) reliability and repeatability for being used to detect small (> 5 mm) lytic lesions, monitor treatment efficacy and damage evolution in longitudinal studies; vi) time efficiency to be implemented in daily practice as part of the standard evaluations instead of being an independent diagnostic procedure; and vii) easiness to apply within a busy clinical practice and applicability to a large number of settings with standard CT available. The agreed requisites for the contents of the MSBDS scoring scale were: i) inclusion of indicators for bone damage; ii) adequate weighing of these items to formulate a scale that most closely reflects the bone involvement in total body CT iii) equal item list for the spine and appendicular skeleton visible on total-body CT.

Within the nominal group technique meetings, interactive sessions examining the total-body CT of MM patients allowed the board members to build-up a tailored methodology for MM evaluation. Scoring of MM lesions was tailored according to previous literature for spinal involvement in oncological patients [6].

A total of 70 MM patients (mean age, 60 years ±9.2 [standard deviation]; range, 35–70 years) undergoing total-body CT with minimum requirements (Table 1) before stem cells transplantations was included to develop MSBDS scores. Patients data were already stored in the Radiological database for other Research studies IRB approved (054/2019).

**Table 1:** Minimal and standard Computed Tomography Technical parameters for inclusion

Number of detector rows*	16 or more up to 128
Minimum Scan coverage*	Skull base to femur
Tube voltage(kV)/time-current product (mAs)	120/50–70, adjusted as clinically needed
Reconstruction convolution kernel	Sharp, high-frequency (bone) and smooth (soft tissue). Middle-frequency kernel for all images are adjusted by the radiologist as deemed necessary
Iterative reconstruction algorithms	Yes (to reduce image noise and streak artefacts)
Thickness	≤5 mm
Multiplanar Reconstructions (MPRs)	Yes (sagittal, coronal and parallel to long axis of proximal limbs)
Matrix, Rotation time, table speed, pith index	128 × 128, 0.5 s,24 mm per gantry rotation, 0.8

The staging and the spectrum of bone lesions were sufficiently broad (Table 2) to give an acceptable coverage of the spectrum of severity of bone involvement. According to our standard procedure, all patients signed a written informed consent form, including the permission to use anonimized data for retrospective research purposes, before CT examination.

**Table 2:** Staging of the 70 MM patients included to develop the MSBDS scores

	Number of patients (*n* = 70)
Durie Salmon Plus	
Grade I	3
Grade II	7
Grade III	51
Grade IV	9
International Staging System	
Grade I	43
Grade II	18
Grade III	9

Readers to test the MSDMS were radiologists and clinicians involved in MM care or expert in bone damage scores (A.T, L.B, A.B.) with different level of experience in musculoskeletal and total body CT namely: reader 1 (A.T.), > 10 years; reader 2 (A.B.), 3 years; reader 3 (L.B.),10 years. Readers were blinded to the clinical data of the patients. A focal lesion relevant for MM was defined as a lesion > 5 mm clinically relevant for diagnosis, prognosis and therapy.

The intra- and inter-observer agreement of the MSBDS score was then calculated. K statistics were used and K values were reported as weighed k with linear weights. 95% confidence intervals (CI) and standard error were also reported. Agreement was defined on the basis of Fleiss classification: < 0.40, poor; 0.40–0.59, moderate; 0.60–0.75, good; > 0.75, excellent [7]. Cronbach’s alpha was used to assess the internal consistency of the method considering values of alpha useful for clinical purposes at least equal to 0.90 [8]. Statistical analysis has been performed with statistical software (MedCalc - version 12.3.0). Finally we correlated MSBDS with MY-RADS score used on CT images to confirm reliability and consistency of the MSBDS compared to MY-RADS.

## Results

The MSBDS scores based on the consensus work described above and literature analysis was finalized as shown in Table 3. MSBDS is based on an additive scale with assessment of a total body CT with the bone window one time and includes indicators of structural bone damage and instability or fracture risk. The total score is given by the sum of item scores for abnormalities detected. Its values range from 0 (minimum) to values > 10 where 10 is represented by high-risk patients. In high-risk patients immediate surgical or radiation oncologist consultation is suggested.

**Table 3:** MSBDS (Myeloma Spine and Bone Damage Score). Interpretation: High-risk: > 10: immediate surgical or radiation oncologist consultation. Medium risk: ≥ 5–10: possible instability and medium risk of pathologic fracture. Low-risk: < 5.

Location	Points
Junctional Spine (C0-C2, C7-T2,T11-L1,L5-S1)	3
Mobile Spine (C3-C6, L2-L4) * only 1 point for semi-rigid (T3-T10)	2
Collapse/involvement > 50%	3
Collapse < 50%*	2
Posterolateral (facet, pedicle) involvement monolateral	2
Posterolateral (facet, pedicle) bilateral monolateral	3
Spinal Canal involvement	5
Trochanteric region focal lesions < 10 mm	2
Femoral neck or entire trochanteric region	5
More 2/3 of bone diameter	3
Focal lesion > 5 mm at any site*	1
Diffuse Pattern	1**

* Bone abnormalities not sufficient to give high risk scores, if isolated. **1 point for every segment according to MY-RADS [9]

**Fig. 1:**
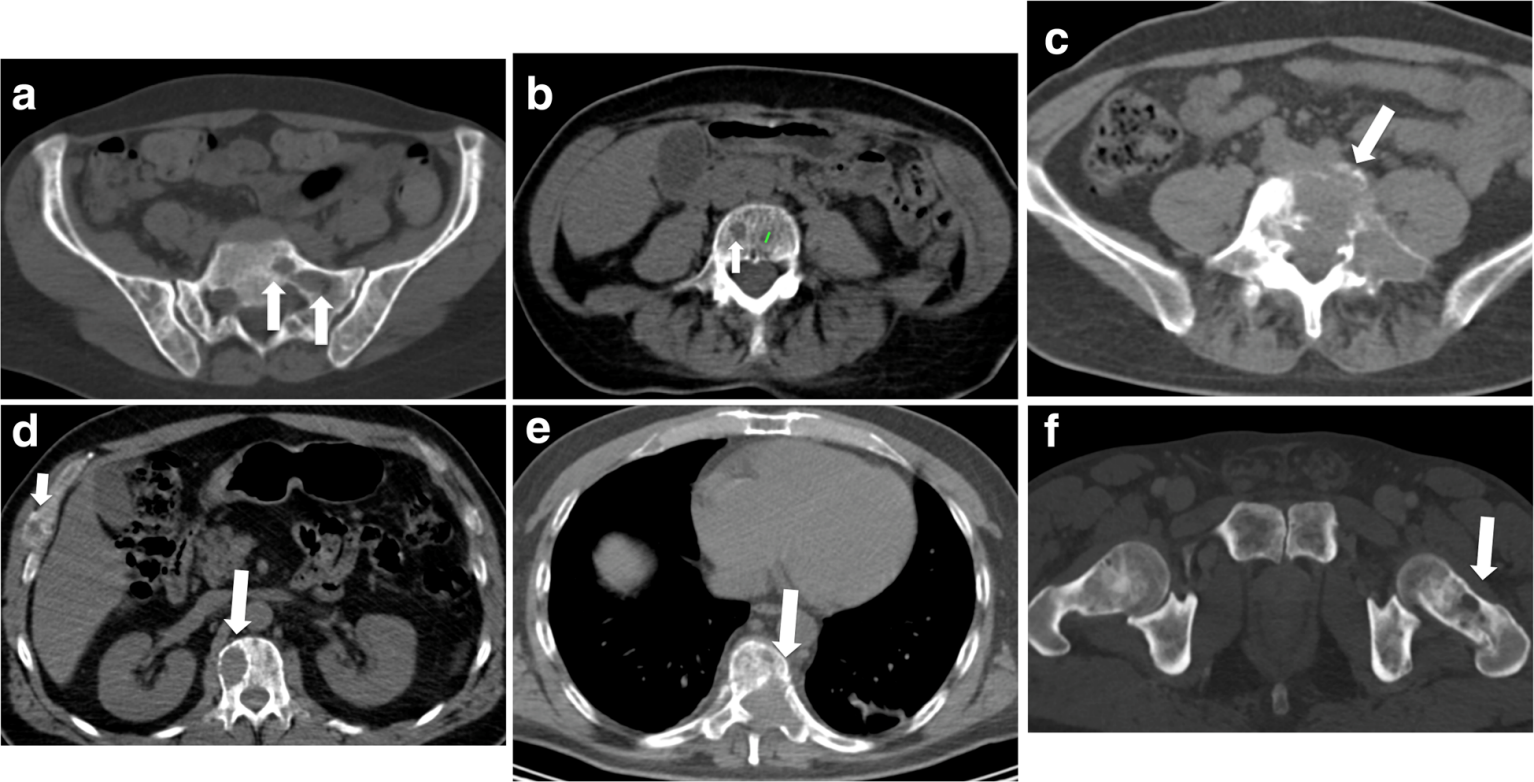
Scoring bone damage and instability - spectrum of findings. **a**) Focal lytic lesions > 5 mm in diameter located at the left sacrum (white arrows). In this case the MSBDS was 2 (1 + 1). **b**) Single focal lytic lesion > 5 mm in the vertebral body (white arrow) with no vertebral collapse. The smaller focal lytic lesion (green line) is < 5 mm (no points in the MSBDS). In this case the MSBDS was 1. **c**) Large lytic lesion at the junctional spine (L5-S1) with collapse/involvement > 50%, posterolateral (facet, pedicle) involvement and more than 2/3 of bone diameter. In this case the MSBDS was 11 (3 + 3 + 2 + 3): the lesion was considered “high-risk” and immediate surgical or radiation oncologist consultation was warranted. In this case, there was also a possible spinal canal involvement. **d**) Lytic lesion > 5 mm (white arrow) at the junctional spine (thoraci spine) with collapse/involvement < 50%, and a small (small white arrow) focal lesion at the right rib cage. In this case the MSBDS was 6 (3 + 2 + 1): the lesion was considered “Medium risk:” 5–10 with medium risk of pathologic fracture. **e**) Large lytic lesion at the junctional spine (thoracic spine) with collapse/involvement > 50%, posterolateral (facet, pedicle) involvement and more than 2/3 of bone diameter. In this case the MSBDS was 11 (3 + 3 + 2 + 3): the lesion was considered “high-risk” and immediate surgical or radiation oncologist consultation was warranted. In this case, there is spinal canal involvement. **f**) Lytic lesion at the left femoral neck (white arrow). This lesion alone warrants 5 points in the MSBDS putting the patients in “medium risk” group

Spectrum of bone findings is reported in Fig. 1a**.**

### Observer agreements

Tables 4 and 5 summarise the analysis of inter- and intra-observer tests. Excellent inter-observer and intra-observer agreement was found among the readers. The Cronbach’s alpha values for all variables were excellent (k = 0.91).

**Table 4:** Inter-observer agreement among the three readers considering the items of the scoring scale using K value, 95% confidence intervals and standard error

	R1-R2	R1-R3	R2-R3
**K**	0.87	0.91	0.85
**95% C.I.**	0.70–0.95	0.78–1.00	0.73–0.98
**SE**	0.07	0.09	0.06

**Table 5:** Intra-observer agreement among the three readers considering the items of the scoring scale using K value, 95% confidence intervals and standard error

	R1	R2	R3
**K**	0.84	0.81	0.85
**95% C.I.**	0.73–0.94	0.73–0.91	0.70–0.93
**SE**	0.09	0.06	0.05

### Correlation with MY-RADS score

A very good correlation between MSBDS and MY-RADS score used on CT images was found: r = 0.816 with *p* < 0.001.

Spectrum of MSBDS score across 70 patients was: high-risk *n* = 17; medium risk *n* = 12; low-risk *n* = 41.

Examples are shown in Fig. 1.

## Discussion

Bone destruction is one of the most prominent features in MM and represent the main target of analysis included in the MSBDS scoring system. The goal of MSBDS is to provide a semi-quantitative tool to evaluate the status of bone damage and risk of fracture and instability in MM. We must emphasize that the evaluation of the status of the bone in MM is important, but it is only one of the many components used to manage MM patient, and perhaps the most difficult component to judge with good agreement among radiologists and clinicians. Indeed, the low agreement between reader in staging patients affected by multiple myeloma is well known in literature [10]: Nanni et al. [9] in the, calculating inter-observer variability with Krippendorff’s alpha, found values of 0.56 to 0.58 indicating only moderate agreement for focal lesions. Data reported in the study by Nanni et al. [9] are consistent with recent results of other studies [11] using total body CT and underlines the need of improvement to correctly evaluate patients with bone involvement. Recently, Radiomics was used to improve focal pattern recognition in MM with promising results [11]: using a Radiomics approach it was possible to increase the accuracy of radiological of focal and diffuse pattern of MM on CT. However, the use of very complex imaging techniques such as Radiomics is not possible to be used routinely and it not possible to be implemented in different working environments at the present time. On the contrary, the use of total body CT could be considered feasible in the majority of centres where stem cells transplantations for MM is available. As for other malignancy, also in multiple myeloma standardization in CT interpretation and scoring of bone lesions is still an unmet need. The evaluation of bone lesions, such as focal lesions, is rapidly gaining importance not only for immediate assessment of dangerous bone lesions (eg. risk of pathological fractures) but also for prognostic purposes. Indeed, the presence of focal lesions is gaining importance because it seems that the presence of focal lesions is linked to poor prognosis [11–15]. In the present study, the MSBDS criteria proved to be highly reproducible, fast and suitable for routine reporting of total body CT in clinical practice in patients with MM undergoing stem cells transplantation. The MSBDS was performed on a relatively high number of patients with relatively advanced stage of disease warranting a high number of bony lesions suitable for the purpose of the study. We demonstrated an “excellent” concordance among the three readers (expert in oncological CT but only marginally trained for MSDBS criteria). This data, as well as the strong correlation with the MY-RADS based score, support the stability of the MSBDS criteria and suggest that this scoring system could be useful to support imaging assessment of total-body CT in MM patients. According to recent guidelines on CT in MM [2, 9, 10, 13–15], the MSDBS represent a significant advance in knowledge because it introduces an easy, feasible and fast to assess semi-quantitatively bone lesions in MM. MSBDS has the advantage that it has been tailored on MM patients, differently from previously published scoring systems previously developed in orthopaedic literature for metastatic patients [6]. Indeed, the MSBDS has been developed not only to assess spinal instability, but also to consider bony involvement for prognostic purposes. The introduction of specific items dedicated to proximal femur involvement and to lytic lesion is typical has the aim to be specific for MM patients. There are several advantages of the MSBDS compared to other scoring systems proposed for MM patients such as the MY-RADS [9]. First, the MSDBS can be used on CT images that are far more available than MR images. Second, the MSDBS is very fast and easily reproducible. We are not aware of thorough data on MY-RADS reproducibility and we believe that MSDBS is easier to be used than MY-RADS. We did not assess bone density on CT because a phantom is usually required to calibrate and we would have introduced another source of variability. The use of Radiomics is complex and introduces several critical factors (starting with the necessity to have homogeneous and calibrated acquisition of CT), however Radiomics is a powerful tool and it will be incorporated in the present model if it will result of real clinical usefulness after careful rigorous evaluation. The presence of a very good correlation between MSBDS and MY-RADS score confirmed the reliability and the consistency of the MSBDS compared to MY-RADS. The MSBDS compared to Durie Salmon and Internatonal Staging System it has not the aim to substitute ISS or Durie Salmon, but it has the aim to improve reports and usage of CT in MM patients. Another advantage of the MSBDS is the relatively low number of analyzed parameters representing a simplification compared to the IMPeTUs criteria for PET or PET/CT. The MSBDS should not increase the burden of data of such a complex disease; therefore we decided to keep this scoring system simple. In addition, the MSDBS could be ready for immediate clinical application indeed it represents a clear improvement of current methods of reporting bone involvement in MM. The MSDBS did not scored different patterns other than focal lesions and diffuse pattern because it seems not clear how other bony patterns visible on CT are linked to prognosis or to the risk of fracture development. It is possible that with the advent of dual-energy CT different radiological patterns will be confidently determined [16]. Moreover, MSBDS should represent an important tool in order to correctly evaluate the patient’s impairment in the medico legal field (e.g.: private health insurances). A prospective clinical validation of MSBDS criteria is underway. We are aware that MSBDS could also be applied in patients with MM even if they are not undergoing stem cells transplantations.

## Conclusions

In this work we have presented the MSBDS descriptive criteria which are easy, highly reproducible and can be considered as a strong base for harmonizing total body CT interpretation in multiple myeloma patients undergoing stem cell transplantation.

## Data Availability

The datasets used and/or analyzed during the current study are available from the corresponding author on reasonable request.

## Abbreviations

MSBDS: Myeloma Spine and Bone Damage Score
MM: Multiple myeloma
SMM: Smouldering multiple myeloma
MRI: Magnetic resonance imaging
CT: Computed tomography
PET/CT: Positron emission tomography–computed tomography
WBLDCT: Whole-body low-dose CT
MY-RADS: Myeloma Response Assessment and Diagnosis System
IMPeTUs: Interpretation criteria for FDG PET/CT in multiple myeloma
